# Utilization of steerable sheath improves the efficiency of atrial fibrillation ablation guided by robotic magnetic navigation compared with fixed‐curve sheath

**DOI:** 10.1002/clc.23801

**Published:** 2022-02-23

**Authors:** Qingzhi Luo, Yun Xie, Yangyang Bao, Yue Wei, Changjian Lin, Ning Zhang, Tianyou Ling, Kang Chen, Wenqi Pan, Liqun Wu, Qi Jin

**Affiliations:** ^1^ Department of Cardiovascular Medicine, Ruijin Hospital Shanghai Jiao Tong University School of Medicine Shanghai China

**Keywords:** atrial fibrillation, catheter ablation, magnetic navigation, steerable sheath

## Abstract

**Background:**

The objective of this study was to assess the impact of steerable sheaths compared with fixed‐curve sheaths on the procedural outcomes of atrial fibrillation (AF) ablation guided by robotic magnetic navigation (RMN).

**Methods and Results:**

In this retrospective case−control study, 110 patients scheduled for AF catheter ablation were enrolled and divided into two groups. Fifty‐five patients (paroxysmal, 70%) were treated with RMN‐guided ablation utilizing a steerable sheath and another 55 patients (paroxysmal, 70%) were ablated with RMN using a fixed‐curve sheath. Clinical characteristics were similar between the two groups. Compared with the fixed‐curve sheath group, the steerable sheath group procedure time (111.9 ± 25.2 vs. 90.4 ± 20.7 min, *p *< .001) and radiofrequency (RF) time (35.9 ± 9.0 vs. 30.5 ± 7.4 min, *p *< .001) were significantly shortened. Additionally, the navigation index was significantly improved (0.41 ± 0.06 vs. 0.48 ± 0.08, *p *< .001) in the steerable sheath group. By employing a large catheter loop for targeting the right pulmonary veins (PVs), the steerable sheath group significantly reduced the RF delivery time (15.0 ± 3.0 vs. 12.0 ± 2.1 min, *p *< .001) during right‐side PV isolation (PVI). However, total fluoroscopy time was similar between the two groups (5.6 ± 2.6 vs. 5.0 ± 2.0 min, *p *> .05). Acute PVI success rates were similar between the two groups. No major or minor complications occurred in either group.

**Conclusion:**

Appropriate utilization of steerable sheath technology can improve the efficiency of AF ablation guided by RMN, primarily by reducing the total procedure and RF delivery times of right‐side PVI without compromising safety.

## 1 INTRODUCTION

Atrial fibrillation (AF) is the most common cardiac arrhythmia in clinical practice and catheter ablation of AF aiming for pulmonary vein isolation (PVI) has emerged as the standard of care once an invasive treatment is indicated.^1^, ^2^ Solid evidence has demonstrated that robotic magnetic navigation (RMN)‐guided AF ablation not only provides increased comfort and associated sustained patient focus for physicians but also has comparable efficacy, superior safety with less peri‐procedural complications, and shorter fluoroscopy time when compared with manual catheter ablation.^3^, ^4^, ^5^ The highly flexible shaft of the magnetic catheter allows for multiple omni‐directional angles of approach. However, maintaining stable contact throughout the entire PVI procedure remains challenging, with adjustments to the sheath position and deflection during the procedure often required. Previous studies have shown that steerable sheath technology has emerged as a means to optimize catheter−tissue contact and improve catheter guidance into different cardiac structures beyond fixed‐curve sheaths.^6^, ^7^, ^8^ However, the utilization of steerable sheaths is associated with increased potential for complications given their rigidity and wider outer diameter, as well as significant incremental cost. Until now, there has been insufficient data on the advantages of applying steerable sheaths in RMN‐guided AF ablation. The purpose of this study was to compare the procedural efficiency parameters, acute PVI success rate, and complication rate using steerable and fixed‐curve sheaths during AF catheter ablation.

## 2 METHODS

### 2.1 Clinical characteristics

In this retrospective study, 55 patients AF refractory to at least one antiarrhythmic agent were ablated with RMN using a steerable sheath. We additionally identified 55 patients previously treated with RMN using a fixed‐curve sheath, who had matching age, gender, and type of AF characteristics. All 110 patients were treated between January 2020 and May 2021. This study was approved by the institutional committee on human research. According to institutional guidelines, all patients provided written informed consent.

### 2.2 Ablation procedure

**Figure 1:**
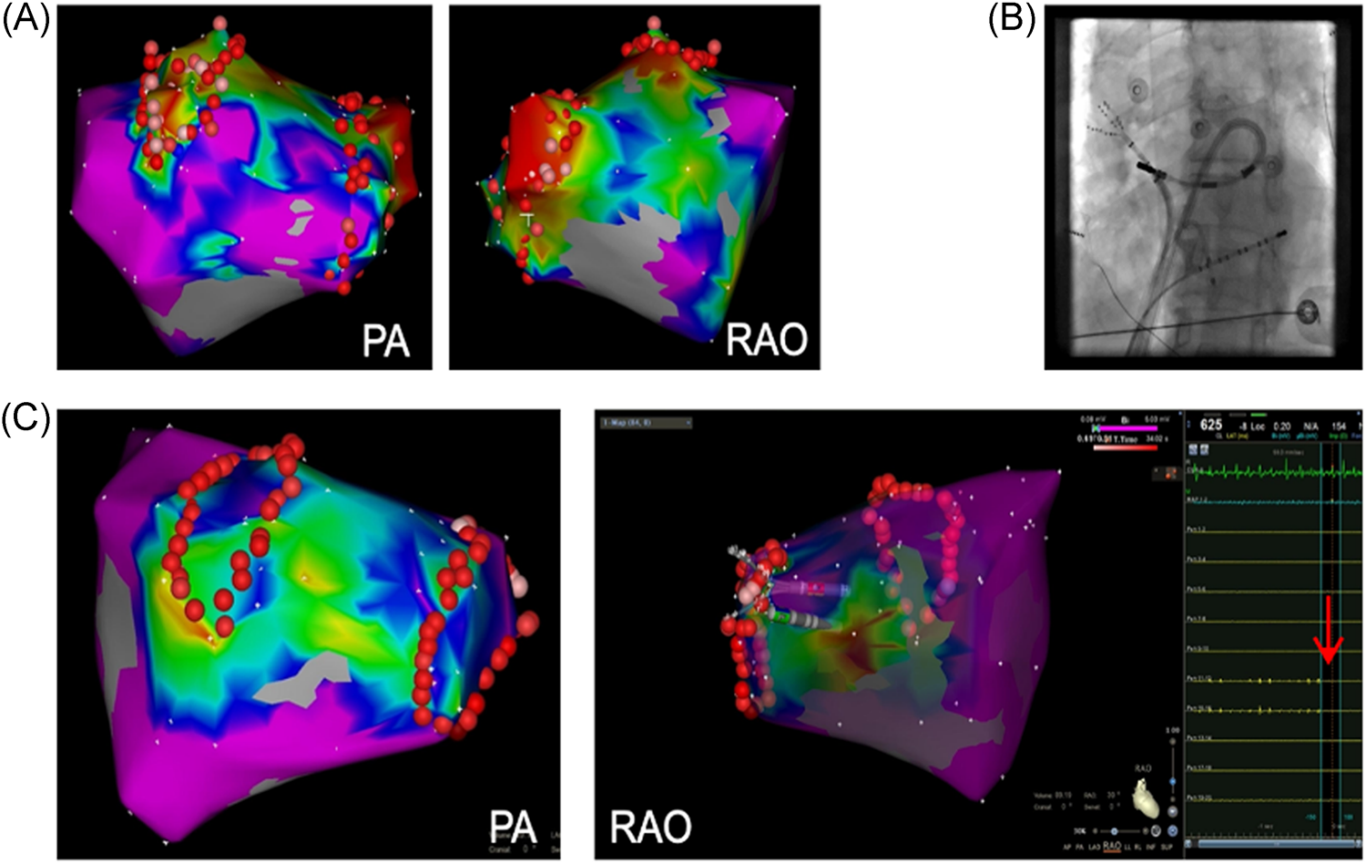
The utilization of steerable sheath facilitates right‐side PVI. (A) A case from the fixed‐curve sheath group in postanterior (PA) and right anterior oblique (RAO) views in the CARTO system. (B) The ablation catheter along with the steerable sheath forming a large loop in the LA to target the RPVs, shown in the LAO view. (C) A case from the steerable sheath group in the CARTO system, in which RF applications are more continuous compared with (A), and the completion of the encircling line results in simultaneous PV isolation (red bold arrow). LA, left atrial; PV, pulmonary vein; PVI, pulmonary vein isolation; RF, radiofrequency; RPV, right pulmonary vein

All patients received uninterrupted anticoagulation therapy with warfarin (target international normalized ratio, INR, 2–3) or direct oral anticoagulants (rivaroxaban or dabigatran) for at least 3 weeks before their procedure. Transoesophageal echocardiography was performed before the procedure to exclude LA thrombus. A steerable 10‐pole catheter (Inquiry, St. Jude Medical, Inc.) and a flexible quadripolar catheter (St. Jude Medical, Inc.) were positioned within the coronary sinus and at the apex of the right ventricle via the left femoral vein, respectively. Two separate transseptal punctures were performed under fluoroscopy. Immediately after the first transseptal puncture, a bolus of 50−100 IU/kg heparin was administered. Activated clotting time (ACT) was rechecked every 30 min during the procedure. Additional heparin boluses were given if necessary to maintain the ACT between 250 and 300 s. A 22‐pole high‐density mapping catheter (Pentaray, Biosense Webster Inc.) to guide PVI was introduced through a long sheath (Fast‐Cath SL1, St. Jude Medical Inc.) and a 3.5 mm tip irrigated magnetic catheter (NaviStar™ RMT ThermoCool™, Biosense Webster Inc.) within a second long sheath. The second long sheath was steerable sheath (MobiCath, Biosense Webster Inc.) in the steerable sheath group or a standard fixed‐curve long sheath (Fast‐Cath SR0, St. Jude Medical Inc.) in the fixed‐curve sheath group. The open‐irrigated ablation catheter was controlled using the CARTO RMT system and RMN Niobe ES system (Stereotaxis Inc.) to perform 3D LA electroanatomic mapping and ablation. All patients underwent circumferential PVI guided by Pentaray with the confirmed endpoint of the entrance block, and additional pacing at multiple points on the multipolar catheter within PVs was performed to check for exit block.^9^, ^10^ PV potentials were distinguished from far‐field potentials with pacing techniques from right atrial or LA appendage (LAA). If AF persisted after PVI, ablation of fractionated electrograms and application of complete lines were performed at the physician's discretion. Looping of the steerable sheath inside the LA was systematically performed to allow a more direct angle of approach to the right PV ostia (from left to right) during right‐side PVI (Figure 1).

### 2.3 Definition of procedural parameters of RMN‐guided ablation

Procedure time was defined as the total time from the Navigant™ “Open Procedure” to the Navigant “Close Procedure” (in minutes). Clinical start time was annotated as the earliest of either the time at which the catheter was registered in the CARTO™ 3D mapping system or the time of first applied magnetic field with RMN. Clinical time was calculated as the time elapsed between clinical start time and the latter time of either the last RMN applied field or the last RF ablation application turned off. Mapping time was the time interval from clinical start time to first burn. Control room X‐ray time was calculated as the total time the fluoroscopy beam was activated while the MNS was in the navigate position. Total X‐ray procedure time was defined as the sum total number of minutes the fluoroscopy beam was activated. RF applications and RF time reflected the total sum of the number and minutes of ablation burns during the procedure, respectively. Ablation time was calculated as the time difference between the first and last RF application times. Navigation index, defined as the ratio of total radiofrequency delivered (in minutes) to the time elapsed from the first burn to the last burn, was utilized to indicate the efficiency of RMN‐guided ablation in this study. The higher the navigation index, the greater percentage of procedure time was spent delivering RF treatment versus locating or navigating to desired RF treatment locations.

### 2.4 Complications

Complications were divided into two categories: minor and major. Minor complications were defined as pericarditis and inguinal hematoma. Major complications included major bleeding, cardiac tamponade, acute myocardial infarction, stroke, atrial‐esophageal fistulae, severe PV stenosis, and procedure‐related death.

### 2.5 Statistical analysis

All continuous variables are expressed as mean ± SD. Two‐tailed *t *tests were used to compare continuous variables and categorical variables were compared by use of the *χ* ^2^ test (or Fisher's exact if *χ* ^2^ test was inappropriate). A two‐tailed *p *< .05 was considered statistically significant. All statistical analyses were performed using SPSS (IBM Corp.).

## 3 RESULTS

### 3.1 Patient characteristics

A total of 110 patients were included in our study. The steerable sheath group and fixed‐curve sheath groups included 55 patients (64.9 ± 11.4 years, 55% male, 70% paroxysmal AF) and 55 patients (62.7 ± 11.2 years, 55% male, 70% paroxysmal AF), respectively. Baseline demographic and clinical characteristics of the two groups are detailed in Table 1. There were no statistically significant differences between the two groups.

**Table 1:** Baseline characteristics

Characteristic	Steerable sheath group	Fixed‐curve sheath group	*p* value
Age (years)	64.9 ± 11.4	62.7 ± 11.2	>.05
Sex (male, %)	30 (55)	30 (55)	>.05
Type of AF (paroxysmal, %)	39 (70)	39 (70)	>.05
BMI (kg/m^2^)	25.3 ± 3.1	24.9 ± 3.8	>.05
LAD (mm)	40.8 ± 3.8	40.4 ± 4.1	>.05
LVEF (%)	63.4 ± 8.0	66.9 ± 3.9	>.05
CHA_2_DS_2_‐VASc score	2.2 ± 1.3	2.3 ± 1.5	>.05
LA volume (ml)	102.9 ± 35.7	102.8 ± 30.9	>.05

Abbreviations: AF, atrial fibrillation; BMI, body mass index; LA, left atrial; LAD, left atrial diameter; LVEF, left ventricular ejection fraction; PV, pulmonary vein.

### 3.2 Ablation procedure outcomes

Procedural parameters are summarized in Table 2. Procedure time was significantly shorter in the steerable sheath group versus the fixed‐curve sheath group (90.4 ± 20.7 vs. 111.9 ± 25.2 min, respectively, *p *< .001). Both mapping time and ablation time were markedly reduced by steerable sheath use (9.9 ± 2.2 vs. 13.1 ± 5.1 min, *p *< .001; 64.4 ± 20.6 vs. 79.5 ± 24.7 min, *p *< 0.001, respectively). RF time was delivered for a shorter time with the utilization of steerable sheath (30.5 ± 7.4 vs. 35.9 ± 9.0 min, *p *< .001). Total fluoroscopy time was similar between the two groups (5.0 ± 2.0 vs. 5.6 ± 2.6 min, *p *> .05). The fixed‐curve group had an underperforming navigation index value compared with that of the steerable sheath (0.41 ± 0.06 vs. 0.48 ± 0.08, *p *< .001), indicating that the steerable sheath might allow for more precise distal tip micro‐movements and increased catheter stability, allowing the catheter to reach the target location more easily.

**Table 2:** Procedural parameters

Parameters	Steerable sheath group	Fixed‐curve sheath group	*p* value
Procedure time (min)	90.4 ± 20.7	111.9 ± 25.2	<.001
Clinical time (min)	75.1 ± 20.8	93.5 ± 24.9	<.001
Total X‐ray time (min)	5.0 ± 2.0	5.6 ± 2.6	.16
Control room X‐ray time (min)	1.9 ± 1.2	1.6 ± 0.9	.17
RF applications (*n*)	80.0 ± 21.3	90.2 ± 23.5	.019
RF time (min)	30.5 ± 7.4	35.9 ± 9.0	<.001
Mapping time (min)	9.9 ± 2.2	13.1 ± 5.1	<.001
Ablation (min)	64.4 ± 20.6	79.5 ± 24.7	<.001
LPVI time (min)	18.5 ± 5.4	20.9 ± 6.0	>.05
RPVI time (min)	12.0 ± 2.1	15.0 ± 3.0	<.001
Navigation index	0.48 ± 0.08	0.41 ± 0.06	<.001

Abbreviations: RF, radiofrequency; LPVI, left pulmonary vein isolation; RPVI, right pulmonary vein isolation.

To further assess the cause of optimized procedure parameters in the steerable sheath group, we compared the ablation of left and right PVs (LPVs and RPVs) between the two groups. On the left side, sheath positioning was similar in both groups. We slightly adjusted the curve of the steerable sheath to more directly orient the ablation catheter to the LPVs. Although utilization of the steerable sheath seemed to make the RF lesions more continuous in many cases, especially on the ridge side (Figure 1A), there was no significant difference in RF delivery time (18.5 ± 5.4 vs. 20.9 ± 6.0 min, *p *> .05) during ablation of the LPVs between the two groups.

When ablating RPVs using a fixed‐curve sheath, the catheter could become unstable in locations such as the inferior aspect of the right inferior PV (RIPV) and the carina between the upper and lower RPVs (Figure 1A). To better target the right PV ostia, the ablation catheter along with the steerable sheath was formed as a large loop inside the LA (Figure 1B). First, we ensured that a sufficient length of the magnetic catheter was outside the sheath to serve as a soft leader guide. Second, we rotated the sheath and advanced laterally in the LA, while simultaneously adjusting the magnetic navigation vector to point septally, effectively looping the sheath and advancing the catheter to more directly approach the RPVs. The utilization of steerable sheaths significantly reduced the ablation time and RF delivery time (12.0 ± 2.1 vs. 15.0 ± 3.0 min, *p *< .001) during right‐side PVI, suggesting an improvement of catheter stability with the steerable sheath, which could prevent the need for additional lesions in such unstable areas (Figure 1C).

### 3.3 Acute procedural success and complications

Acute procedural success did not differ significantly between the two groups (steerable sheath 100% vs. fixed‐curve sheath 100%). There were no minor or major complications observed in either group.

## 4 DISCUSSION

### 4.1 Main findings

In this study, we reported our initial experience of steerable sheaths used in AF ablation guided by RMN. Compared with the fixed‐curve sheath group, the mapping time and ablation time were significantly reduced in the steerable sheath group. Our observation is that this was likely due to improved catheter stability as evidenced by the improved navigation index, thereby shortening procedure time. Right‐side PVI was faster and RF delivery time was shorter when a steerable sheath was used. Comparable acute success and safety performance were seen between the two groups.

### 4.2 Advantages of RMN‐guided ablation

When compared with manual‐guided navigation, RMN‐guided catheter ablation in AF offers the advantages of precise and flexible catheter navigation, reduction in peri‐procedure complications, and fluoroscopy exposure.^11^, ^12^ In this study, the total procedure time of all patients was shorter than that reported in our recent article,^13^ which showed that procedure time decreased along the learning curve from 2010 to 2019. Moreover, all data in this study were derived with the third‐generation RMN system (Niobe ES), providing faster magnetic field direction changes than the Niobe II system, thus reducing procedure and RF times during AF ablation.^14^ Other contributing factors could be the greater percentage of paroxysmal AF patients and smaller LA volumes than those reported in our previous study.^15^

Strong evidence suggests the predictive value of catheter–tissue contact for the depth of ablation lesions. Compared with conventional sheaths, the utilization of steerable sheaths in ablation has been confirmed to increase catheter stability and tissue contact, thus improving ablation outcomes.^7^, ^8^, ^11^ Contact force (CF) sensing technology is not available with RMN, but the “Magnetic Torque Meter,” which measures the angular difference (0−90°) between the applied magnetic field direction and current catheter tip orientation, can be considered a semi‐quantitative contact tool embedded in the RMN system. During AF ablation, operators pay attention to this real‐time measurement and only deliver RF when the meter indicates consistent contact in the desired range. In an experimental model, magnetic fields of 0.08 and 0.10 T provide stable catheter CF, with an average of 6 g without a sheath, which increased to 20 g with a long sheath positioned at the entrance of the chamber of interest.^16^ Moreover, previous studies have suggested that RMN‐guided AF ablation resulted in larger lesion dimensions and faster modification of electrograms when compared with manual‐guided ablation with optimized CF.^17^, ^18^

### 4.3 Best practices of steerable sheath coupled with RMN

The results demonstrate that the navigation index is significantly increased with steerable sheath use, indicating that its employment can significantly improve ablation efficiency when compared with a fixed‐curve sheath. While in RMN‐guided procedures, the fixed‐curve sheath is primarily only used to provide the catheter entry into the chamber. In this study, the steerable sheath was used to provide an anchoring point opposite the ablation target site to optimize the angle of approach and improve catheter tip stability. For example, when ablating LPVs, we regularly advanced the steerable sheath toward the LPVs and slightly anteriorly if the body of the steerable sheath was oriented toward the posterior wall. For ablation along the ridge between LPVs and LAA, where the catheter was often unstable due to unintentional catheter slippage on the ridge of the LAA or PV ostium, this orientation provided the desired catheter stability (Figure 1). However, the RF delivery time was not significantly reduced in this area. When ablating RPVs, we regularly advanced the sheath laterally and superior inside the LA, and then pointed septally to form a large loop for targeting RPVs (Figure 1). Thus, a longer length of magnetic catheter is available with all three catheter magnets outside the sheath, providing omni‐directional steerability and stable contact. With a fixed‐curve sheath, we focus on optimizing transseptal puncture location to more easily access the RPVs, especially in patients with smaller atria.^19^ We note that with the steerable sheath optimizing transseptal location for RPV access is less critical and this could be helpful for centers without intracardiac echocardiography available for transseptal access. When using the fixed‐curve sheath, in some patients additional ablation points might need to be applied on the carina between the upper and lower PVs to achieve PVI after completion of the encircling line. However, the utilization of a steerable sheath could reduce the incidence of applying additional RF lesions on the carina, particularly during right‐side PVI, thus decreasing RF delivery time (Figure 1).

### 4.4 Study limitations

The major limitation of this study is that it is a nonrandomized retrospective case−control study in a single center. The patient population was matched by baseline characteristics and the ablation data were pooled from the same physician. These factors should have partially minimized bias from the nonrandomized design of this study. However, a further prospective large‐scale multicenter study is needed to confirm the effect of the utilization of steerable sheath technology with the RMN system.

## 5 CONCLUSION

The utilization of steerable sheath technology with RMN for AF catheter ablation significantly reduced the procedure time, while achieving comparable acute success without compromising safety, when compared with the use of a conventional fixed‐curve sheath. Looping of the steerable sheath inside the LA facilitated right‐side PVI navigation and reduced RF delivery time by providing more consistent, stable contact.

## CONFLICT OF INTERESTS

The authors declare no conflict of interests.

## Data Availability

The data that support the findings of this study are available on request from the corresponding author. The data are not publicly available due to privacy or ethical restrictions.
